# Are post-treatment low-density lipoprotein subclass pattern analyses potentially misleading?

**DOI:** 10.1186/1476-511X-9-136

**Published:** 2010-11-30

**Authors:** Harold Bays, Scott Conard, Lawrence A Leiter, Steven Bird, Erin Jensen, Mary E Hanson, Arvind Shah, Andrew M Tershakovec

**Affiliations:** 1 Louisville Metabolic and Atherosclerosis Research Center, Louisville, KY, USA; 2 Medical Edge Healthcare Group, P.A., Dallas, TX, USA; 3 Keenan Research Centre in the Li Ka Shing Knowledge Institute of St. Michael's Hospital and the University of Toronto, Toronto, ON, Canada; 4 Merck, Sharp & Dohme Corp., a div. of Merck & Co., Inc., Whitehouse Station, NJ, USA

## Abstract

**Background:**

Some patients administered cholesterol-lowering therapies may experience an increase in the proportion of small LDL particles, which may be misinterpreted as a worsening of atherosclerotic coronary heart disease risk. This study assessed the lipid effects of adding ezetimibe to atorvastatin or doubling the atorvastatin dose on low-density lipoprotein cholesterol (LDL-C) levels (and the cholesterol content of LDL subclasses), LDL particle number (approximated by apolipoprotein B), and LDL particle size. This was a multicenter, double-blind, randomized, parallel-group study of hypercholesterolemic, high atherosclerotic coronary heart disease risk patients. After stabilization of atorvastatin 40 mg, 579 patients with LDL-C >70 mg/dL were randomized to 6 weeks of ezetimibe + atorvastatin 40 mg or atorvastatin 80 mg. Efficacy parameters included changes from baseline in LDL-C, apolipoprotein B, non-high-density lipoprotein cholesterol (non-HDL-C), and lipoprotein subclasses (Vertical Auto Profile II) and pattern for the overall population, as well as patient subgroups with baseline triglyceride levels <150 mg/dL or ≥150 mg/dL.

**Results:**

Both treatments significantly reduced LDL-C (and the cholesterol content of most LDL subfractions [LDL_1-4_]) apolipoprotein B, non-HDL-C levels, but did not reduce the proportion of smaller, more dense LDL particles; in fact, the proportion of Pattern B was numerically increased. Results were generally similar in patients with triglyceride levels <150 or ≥150 mg/dL.

**Conclusions:**

When assessing the effects of escalating cholesterol-lowering therapy, effects upon Pattern B alone to assess coronary heart disease risk may be misleading when interpreted without considerations of other lipid effects, such as reductions in LDL-C, atherogenic lipoprotein particle concentration, and non-HDL-C levels.

**Trial Registration:**

(Registered at clinicaltrials.gov: Clinical trial # NCT00276484)

## Introduction

Landmark CHD outcomes trials demonstrate that, in general, LDL-C lowering therapies reduce CHD risk. Statin-treated patients who achieve greater LDL-C lowering (either through an increase in the same statin dose or through use of a different statin) have reduced CHD events compared with statin-treated patients with less LDL-C lowering[1-3]. In addition, non-high-density lipoprotein cholesterol (non-HDL-C) and apolipoprotein B (apo B) may be better predictors of CHD risk than LDL-C levels[4]. Non-HDL-C is a measure of the cholesterol carried by all atherogenic lipoproteins, such as the cholesterol carried by LDL particles, as well as the cholesterol carried by very low-density lipoproteins, intermediate-density lipoproteins, remnant lipoproteins, chylomicrons (and their remnants), and lipoprotein (a). Regarding particle number, one apo B molecule is found on each lipoprotein particle; thus, apo B level is often considered a surrogate marker for atherogenic lipoprotein particle concentration. An increase in atherogenic lipoprotein particle number is thought to increase CHD risk[5,6].

Lipoprotein particle size is another lipid parameter that may influence CHD risk. A disproportionate increase in smaller LDL particles is often described as increasing CHD risk[7]. However, it is unclear whether LDL particle size provides additional predictive power for measuring CHD risk versus LDL particle number[8]. Various commercial analyses are available for additional lipid testing, sometimes described as "advanced" lipid testing. One example is vertical auto profile (VAP), which is a direct ultracentrifugation method that uses vertical rotor and single density gradient spin[9]. According to VAP II analyses (Atherotech, Inc., Birmingham, AL, USA), low-density lipoprotein particles are reported as four subclasses based on density. The larger, more buoyant LDL particles are LDL_1 _and LDL_2, _and the smaller, denser particles are LDL_3 _and LDL_4_. VAP analyses also report the cholesterol carried by each lipoprotein subclass. An increase in the proportion of smaller, more dense LDL particles is referred to as Pattern B, which is considered to impart greater CHD risk[10-14]. HDL-C is a highly heterogeneous lipoprotein that can be separated into two major subclasses (HDL-C_2 _and HDL-C_3_) and several minor subclasses based on density. Both major subclasses are inversely related to CHD risk,[11,15] and low HDL-C and low HDL particle concentration are associated with increased risk for CHD[16].

Mechanistically, the proposed increased atherogenicity of the smaller, more dense LDL particles may be related to their decreased affinity for tissue and liver LDL receptors, which leads to prolonged LDL particle presence in the blood, and thus increased exposure to aterioles[17]. In addition, the small, more dense LDL particles may have increased permeability through the arterial endothelium and may be preferentially retained in the arterial wall[18]. Moreover, these particles may be more readily oxidized, further increasing their atherogenic potential[19].

A practical, clinical challenge regarding advanced lipid testing is that the use of lipoprotein pattern analysis for pre-treatment diagnostic purposes may have very different clinical implications than the use of lipoprotein pattern analysis to assess the efficacy of cholesterol-lowering therapy. Some clinicians believe that the presence of pre-treatment Pattern B confers an increased CHD risk, thus prompting them to be more aggressive with lipid-altering therapy. But anecdotally, some clinicians also believe that a post-treatment shift to Pattern B likewise increases CHD risk, which may prompt them to consider discontinuing or altering cholesterol-lowering therapy. This is of clinical importance given the wealth of data supporting LDL-C lowering as reducing CHD events [20] and the lack of CHD outcome data supporting the "improvement" in lipoprotein particle size as reducing CHD events.

This study analyzed the effects of ezetimibe added to atorvastatin 40 mg or doubling the atorvastatin dose in atorvastatin-treated, hypercholesterolemic, high CHD risk patients [21]. Efficacy parameters included LDL-C levels, the cholesterol content of LDL and HDL subclasses, apo B, non-HDL-C levels and LDL particle size (Pattern). In addition, the same endpoints were assessed in a *post hoc *analysis of subgroups of patients with baseline triglyceride levels <150 mg/dL (normal) or ≥150 mg/dL (elevated), since these levels approximate the triglyceride threshold by which LDL particle size is most likely to shift to larger or smaller LDL particles[22].

## Methods

The methods of this study were previously published[21]. Briefly, this was a multicenter, double-blind, randomized, parallel-group study conducted at 96 sites in the US (91) and Canada (5), from April 2006 to February 2008, conducted under Good Clinical Practices guidelines. The study protocol underwent review and approval by institutional review boards and study participants provided written informed consent prior to study procedures being performed. Entry criteria included hypercholesterolemic adults <80 years who had CHD, a CHD risk equivalent medical condition, or 2 or more CHD risk factors and a Framingham Risk Score estimating a 10-year risk for CHD >20%[23].

Other entry criteria included triglyceride levels ≤350 mg/dL, hemoglobin A1c <8.5%, liver transaminases (alanine aminotransferase [ALT] and aspartate aminotransferase [AST]) ≤1.5 X the upper limit of normal (ULN) with no active liver disease, and creatinine kinase (CK) levels ≤2 X ULN. Patients must have received a stable daily dose of a statin of equal or lesser LDL-C lowering efficacy than atorvastatin 40 mg/d, or must have been naïve to statin, ezetimibe, or ezetimibe/simvastatin therapy. Exclusion criteria included patients taking other prescription and/or over-the-counter-drugs/supplements with the potential for significant lipid-altering effects or therapies with the potential for drug interactions with atorvastatin.

### Treatments

Patients entering the study agreed to follow the National Cholesterol Education Program therapeutic lifestyle changes/American Diabetes Association or similar cholesterol-lowering diet throughout the trial. Patients already taking atorvastatin 40 mg/d at study entry continued this therapy for a 4-week run-in period. Those taking a statin with equal or lower LDL-C lowering efficacy and those naïve to lipid-altering drug therapy received atorvastatin 40 mg/d for a 5-week run-in period. Following the run-in period, patients were randomized to 6 weeks of atorvastatin 40 mg/d plus ezetimibe (10 mg) or atorvastatin 80 mg/d.

### Efficacy endpoints

In addition to efficacy endpoints in the overall population, which included all randomized patients who took at least one dose of study medication and had a baseline value and at least one post baseline value, this analysis also evaluated treatment efficacy at week 6 by subgroups based on normal and elevated baseline triglycerides (<150 mg/dL or ≥150 mg/dL). A central laboratory (PPD, Highland Heights, KY, USA) was utilized, which measured common lipid parameters, including apo B and safety laboratory values. For patients with triglycerides ≤400 mg/dL, LDL-C measurements were calculated by the Friedewald equation. For patients whose triglycerides may have increased to >400 mg/dL during the study, LDL-C measurement was obtained directly using beta quantification. VAP II was the method used to measure lipoprotein particle size, cholesterol content of the LDL-C_1-4 _lipoprotein subclasses, and lipoprotein pattern[9,24].

The VAP II method defines the LDL pattern based on the value of the LDL max time. Lower LDL max times (≤115 seconds) correspond to predominantly small and dense LDL (Pattern B), and higher LDL max times (≥118 seconds) correspond to predominantly large and buoyant LDL particles (Pattern A). Patients with LDL max times between 115 and 118 seconds are identified as having intermediate pattern (Pattern A/B or Pattern I)[9].

### Statistics

The statistical analyses for the traditional lipid parameters were previously described[21]. Treatment group comparisons of interest were the same as the primary study comparison (atorvastatin 40 mg + ezetimibe or atorvastatin 80 mg). Subgroup analysis of patients with normal (<150 mg/dL) and elevated (≥150 mg/dL) baseline triglyceride levels utilized nonparametric methods for median percent change from baseline for LDL subclasses 1-4. Determination of mean percent change from baseline in total LDL-C, apo B, and non-HDL-C utilized an analysis of covariance model with terms for treatment, baseline variable, triglyceride subgroup, and treatment by triglyceride subgroup interaction. Additionally, the categorical distribution of R-LDL pattern (A, I, or B) at baseline and study end was summarized in the overall population and for both the normal and elevated baseline triglyceride groups using the Cochran-Mantel-Haenszel Chi-square statistic. For all of the analyses performed on the subgroups of patients with normal (<150 mg/dL) and elevated (≥150 mg/dL) baseline triglyceride levels, no inferential statistics were conducted to avoid issues of multiplicity, although 95% confidence intervals were calculated.

To provide perspective on interpreting results for multiple exploratory endpoints and to minimize the likelihood of falsely identifying a significant treatment difference, the false discovery rate (FDR) procedure [25] was applied to the prespecified lipoprotein subclass analyses for the overall population. The FDR procedure was applied at the 0.05 level and included 8 parameters for the LDL family of lipoproteins: LDL (Total), LDL (Real), R-LDL subclass pattern, LDL-R peak max time, LDL_1_, LDL_2_, LDL_3_, LDL_4_; and 10 parameters for the HDL family of lipoproteins: HDL-C, HDL_2_, HDL_2a _HDL_2b_, HDL_2c_, HDL_3_, HDL_3a_, HDL_3b_, HDL_3c _and HDL_3d_.

## Results

### Patients

Enrollment and patient flow through the study was previously summarized[21]. Of the 579 patients randomized, the majority were white (81%) and male (60.6%). The mean age was 61 (±10) years.

**Figure 1:**
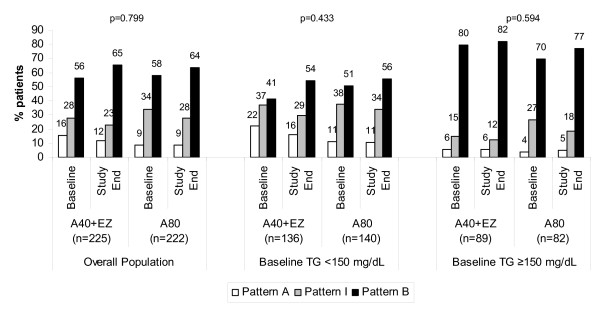
**Change in LDL subclass pattern in the overall population and by baseline triglyceride levels (<150 or ≥150 mg/dL) in high risk patients treated for 6 weeks with ezetimibe added to atorvastatin 40 mg vs atorvastatin 80 mg**. P-values are for between-treatment comparison (Atorva 40 + EZ vs Atorva 80)

Among the overall population, baseline Pattern I and Pattern B were more prevalent than Pattern A (Figure 1). Pattern B was even more disproportionally higher than Pattern A in those with triglyceride levels ≥150 mg/dl (Figure 1).

Total baseline (i.e. while on atorvastatin 40 mg per day) LDL-C levels were 88.6 and 89.7 mg/dL in the overall population and 88.3 and 85.6 mg/dL in patients with normal baseline triglycerides (Table 1). In patients with elevated triglyceride levels, baseline LDL-C levels were 88.9 and 96.1 mg/dL (Table 1). Among the overall population, the individual LDL subclass with the greatest baseline cholesterol content was the small, denser LDL_3 _subclass (Table 1). The cholesterol content in the LDL_3 _subclass was greatest in those with elevated triglyceride levels (Table 1). Among the overall population, baseline apo B was approximately 100 mg/dL, and highest among those with elevated baseline triglyceride levels (Table 1). Finally, baseline non-HDL-C levels were approximately 118 mg/dL in the overall population, 108 mg/dL in patients with normal baseline triglycerides, and highest in patients with elevated baseline triglycerides (ranging from 130.9 to 135.5 mg/dL; Table 1).

**Table 1:** Baseline and study end median values of Apolipoprotein B and cholesterol content in lipoprotein subclasses in the overall population and triglyceride subgroups in high risk patients

(mg/dL)	Overall Population		Baseline Triglycerides <150 mg/dL		Baseline Triglycerides ≥**150 mg/dL**	
**Baseline concentration**	**A40 + EZ n = 225**	**A80 n = 222**	**A40 + EZ n = 136**	**A80 n = 140**	**A40 + EZ n = 89**	**A80 n = 82**

Triglyceride	131.0	135.5	109.0	107.5	195.8	191.0
Apo B*	101.1	102.0	94.8	94.8	111.7	113.7
Total LDL-C*	88.6	89.7	88.3	85.8	88.9	96.1
LDL_1_-C	14.0	14.0	14.0	13.0	15.0	16.0
LDL_2_-C	17.0	16.5	21.0	18.5	10.0	13.5
LDL_3_-C	38.0	41.5	36.0	39.0	42.0	44.0
LDL_4_-C	9.0	9.0	7.0	8.0	14.0	13.0
Non-HDL-C*	117.4	118.0	109.3	107.3	130.9	135.5
HDL_2_-C	9.0	10.0	10.0	10.00	8.0	8.5
HDL_3_-C	36.0	36.0	38.0	36.0	33.0	34.0

**Study end concentration**						

Triglyceride	117.0	124.0	92.0	104.0	166.5	170.0
Apo B*	82.5	93.2	79.3	87.9	87.7	101.8
Total LDL-C*	64.1	79.1	65.3	76.8	62.2	82.8
LDL_1_-C	10.0	12.0	9.0	12.0	11.0	13.5
LDL_2_-C	13.0	15.0	15.0	17.0	10.0	13.0
LDL_3_-C	29.0	37.0	27.0	35.0	30.0	40.0
LDL_4_-C	8.0	9.0	7.0	8.0	10.0	11.0
Non-HDL-C*	89.5	106.4	84.8	99.0	97.5	118.6
HDL_2_-C	10.0	9.5	10.0	10.0	9.0	8.0
HDL_3_-C	35.0	35.0	37.0	36.0	33.0	34.0

*expressed as mean value; n = 277 for A40 + EZ and n = 279 for A80

A = Atorvastatin; C = cholesterol; EZ = ezetimibe 10 mg; HDL = high-density lipoprotein; LDL = low-density lipoprotein

### Lipid effects

#### LDL-C subclass pattern

Among the overall population, neither atorvastatin 40 mg + ezetimibe nor atorvastatin 80 mg increased Pattern A. In fact, both regimens numerically decreased the proportion of patients with Pattern A and Pattern I and increased the proportion of patients with Pattern B compared with baseline (Figure 1). Athough this pattern shift did not differ significantly, and although the prevalence of both baseline and end-of-study Pattern B was higher among those with elevated baseline triglyceride levels, Figure 1 supports a consistency in the direction of this shift in the overall population, as well as in both triglyceride subgroups (Figure 1).

#### LDL-C levels and cholesterol content of LDL subclasses

**Figure 2:**
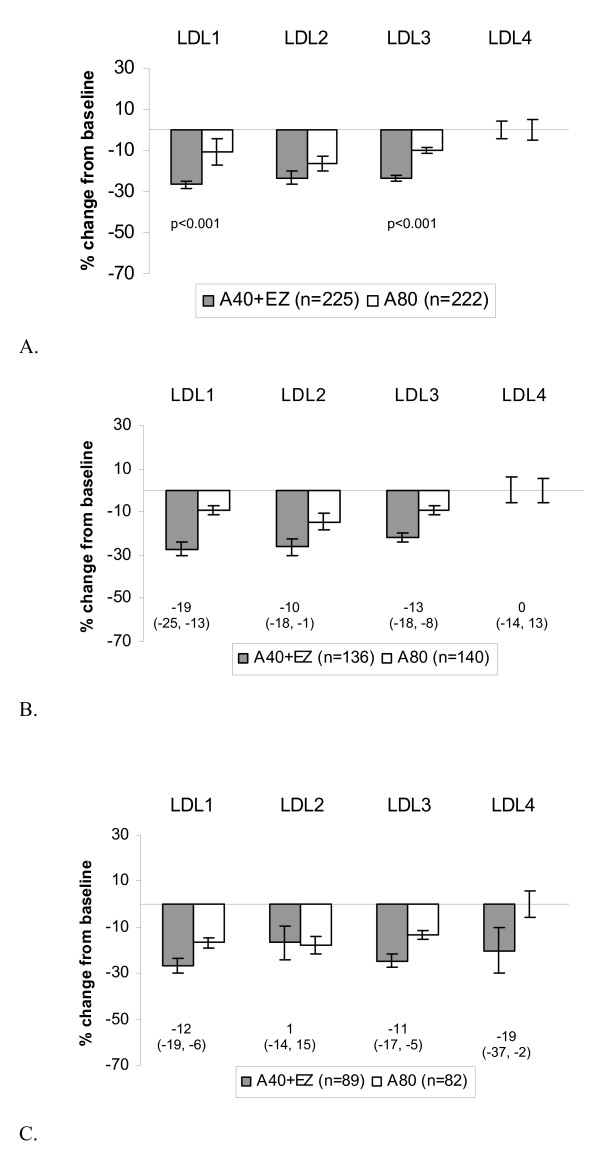
**Median percent change from baseline and between-treatment differences (A40+EZ minus A80) in cholesterol content of lipoprotein subclasses (LDL-C**_**1-4**_**) after treatment with ezetimibe added to atorvastatin 40 mg vs doubling to atorvastatin 80 mg for 6 weeks in the overall population and in subgroups with baseline triglyceride <150 or ≥150 mg/dL**. Numbers below bars in figures B and C represent the between-treatment difference (95% confidence interval). **B**. Baseline triglycerides <150 mg/dL. **C**. Baseline triglycerides ≥150 mg/dL.

Both treatments reduced total LDL-C from baseline (Table 1), with a significantly greater reduction observed in the atorvastatin 40 mg + ezetimibe 10-mg group compared with the atorvastatin 80-mg group in the overall population (-27.4% vs -11.0%, *P *< 0.001). Those with normal baseline triglyceride levels generally had a similar reduction in LDL-C levels compared with those who had elevated triglyceride levels (Table 1). Among the overall population atorvastatin 40 mg + ezetimibe and atorvastatin 80 mg lowered the cholesterol content of most LDL subfractions. The degree of cholesterol lowering in individual subfractions was generally similar with regard to those with normal or elevated triglyceride levels (Table 1 and Figure 2), with the exception of the increased reduction in LDL_4 _for those with elevated baseline triglyceride levels.

Figure 2A illustrates that among the overall population, atorvastatin 40 mg + ezetimibe lowered the cholesterol content of LDL_1 _and LDL_3 _significantly more than atorvastatin 80 mg. (adjusted *P*-values ≤ 0.001). Changes in the cholesterol content of LDL_2 _and LDL_4 _subclasses were similar between treatment groups (adjusted *P*-values > 0.05). No inferential statistics were conducted in triglyceride subgroups; however, among those with normal baseline triglyceride levels (Figure 2B), atorvastatin 40 mg + ezetimibe appeared to reduce the cholesterol content of LDL_1_, LDL_2_, and LDL_3 _subclasses more versus atorvastatin 80 mg. Among those with elevated triglyceride levels (Figure 2C), atorvastatin 40 mg + ezetimibe appeared to reduce the cholesterol content of LDL_1_, LDL_3_, and LDL_4 _subclasses more versus atorvastatin 80 mg.

#### Apo B and non-HDL-C

**Figure 3:**
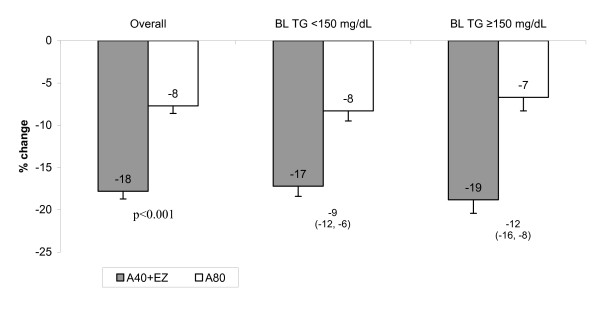
**Mean % change from baseline in apo B after treatment with ezetimibe added to atorvastatin vs doubling the atorvastatin dose for 6 weeks in the overall population and in subgroups with baseline triglycerides (BL TG) <150 or ≥150 mg/dL**. Numbers below bars represent the between treatment difference (95% confidence interval) Overall population, A40 + EZ: n = 277; A80: n = 279; Baseline triglycerides <150 mg/dL, A40 +EZ: n = 136; A80: n = 140; Baseline triglycerides ≥150 mg/dL, A40 +EZ: n = 89; A80: n = 82

Among the overall population, both atorvastatin 40 mg + ezetimibe and atorvastatin 80 mg lowered apo B levels (Table 1; Figure 3). Patients treated with atorvastatin 40 mg + ezetimibe had a significantly greater reduction in apo B levels compared with atorvastatin 80 mg (-17.8% vs. -7.7%, *P *< 0.001: Figure 3). Among the triglyceride subgroups, the mean percent change from baseline was greater in patients treated with atorvastatin 40 mg + ezetimibe compared with atorvastatin 80 mg [normal triglycerides at baseline (-17.2% vs -8.3%) and elevated triglycerides at baseline (-18.8% vs -6.7%)] (Table 1 and Figure 3).

**Figure 4:**
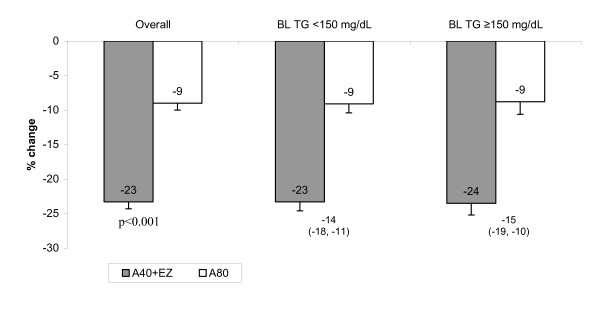
**Mean % change from baseline in non-HDL-C after treatment with ezetimibe added to atorvastatin vs doubling the atorvastatin dose for 6 weeks in the overall population and in subgroups with baseline triglycerides (BL TG) <150 or ≥150 mg/dL**. Numbers below bars represent the between treatment difference (95% confidence interval). Overall population, A40 + EZ: n = 225; A80: n = 222; Baseline triglycerides <150 mg/dL, A40 + EZ: n = 136; A80: n = 140; Baseline triglycerides ≥150 mg/dL, A40 + EZ: n = 89; A80: n = 82

Among the overall population, both atorvastatin 40 mg + ezetimibe and atorvastatin 80 mg lowered non-HDL-C, with a significantly greater reduction in the group treated with atorvastatin 40 mg + ezetimibe compared with those treated with atorvastatin 80 mg (-23.3% vs. -9.0%, *P *< 0.001: Figure 4). The results were similar among the triglyceride subgroups (Figure 4).

#### HDL particle size

In the overall population, the median percent changes from baseline in the HDL_2 _and HDL_3 _subclasses after 6 weeks of treatment with atorvastatin 40 mg + ezetimibe and atorvastatin 80 mg were both 0.0% (robust standard deviations were 28.0 and 27.1, respectively). Similar to the overall study results for HDL_2 _and HDL_3, _neither treatment resulted in substantive changes from baseline after 6 weeks of treatment in either of the triglyceride subgroups (Table 1). For both HDL subclasses, the median percent changes were 0.0% in both treatment groups, regardless of baseline triglyceride levels, except the high triglyceride group treated with atorvastatin 80 mg, which experienced a -2.5% change from baseline in the HDL_3 _subclass.

## Discussion

In this study, both atorvastatin 40 mg + ezetimibe and atorvastatin 80 mg lowered LDL-C levels (as well as the cholesterol content of most LDL subfractions as measured by VAP II methodology), reduced atherogenic lipoprotein particle concentration (as measured by apo B), and reduced non-HDL-C levels. However, treatment with atorvastatin 40 mg + ezetimibe and atorvastatin 80 mg also numerically increased the proportion of patients with LDL subclass Pattern B. Although these changes were not statistically significant, Figure 1 supports a remarkably consistent shift in all studied groups from Pattern A to Patterns I & B, and Pattern I to Pattern B in the overall study group, as well as study participants with TG <150 and ≥150 mg/dL.

Despite the numerous published statin studies, prior reports of the effects of statins on Pattern B are scarce and inconsistent, possibly because of the questionable clinical relevance of this parameter as a post-treatment measure, and the potential misinterpretation of the results. Regarding atorvastatin, a previous, uncontrolled, small trial (n = 26) of hypercholesterolemic patients using nuclear magnetic resonance (NMR) revealed that atorvastatin 10 mg a day significantly lowered LDL-C levels, significantly reduced LDL particle number, significantly reduced the cholesterol content of LDL subclasses (large and small), significantly increased overall LDL particle size, but had no significant effect upon Pattern B[26]. In a larger, placebo-controlled study of 217 dyslipidemic patients with type 2 diabetes mellitus, using polyacrylamide gradient gel electrophoresis, atorvastatin 10 mg and 80 mg significantly lowered LDL-C levels, significantly reduced apo B, and produced no significant effects upon LDL particle size. However, similar to this report, atorvastatin produced a numerical increase in the proportion of patients with Pattern B (21.3%, 21.4%, and 22.2% for placebo, atorvastatin 10 mg, and atorvastatin 80 mg, respectively)[27].

The effects of statin combination therapy upon lipoprotein particle size may be dependent upon the lipid-altering drug used in combination with the statin. Generally, an increase in LDL particle size would be expected with niacin,[28] omega-3-fatty acids[29] or fibrates[30]. Similarly, ezetimibe combined with fenofibrate reduces LDL-C levels, reduces atherogenic lipoprotein particle concentration as measured by a reduction in apo B and reduces non-HDL-C. The proportion of patients with Pattern B in those administered ezetimibe and fenofibrate is reduced as well[31,32]. In prior studies with ezetimibe monotherapy, or ezetimibe combined with statins, LDL particle size was either increased,[33] remained the same,[34] or decreased[35].

In a smaller, single-site study of 72 healthy men involving simvastatin and ezetimibe, lipoprotein particle size (determined by gradient gel electrophoresis) suggested that ezetimibe alone or in combination with simvastatin increased small, dense LDL particles. The authors concluded: *"In healthy men, treatment with ezetimibe alone is associated with the development of a pro-atherogenic LDL subfraction profile. Potentially atheroprotective effects of simvastatin are offset by ezetimibe." *[36] However, the authors acknowledged that statin, ezetimibe, and the combination of ezetimibe and statin all "*decreased the large, more buoyant LDL-I subfraction." *To the extent that this reflects a decrease in the number of atherogenic particles, then this would seem to be a favorable lipid effect. Furthermore, this single-site study was not a CHD outcomes study. It is therefore unclear how the authors' data support their claim that the atheroprotective effects of statins are "offset" by ezetimibe, based upon their reported proportional effects on lipoprotein particle size alone, and irrespective of effects upon LDL-C, non-HDL-C, and apo B levels and especially given the lack of objective atherosclerosis data in their small, single-site study. This is potentially an illustrative example of how advanced lipid testing results might be misinterpreted when considered outside the context of other lipid effects, such as reductions in LDL-C levels and reductions in particle number (apo B), which (as previously described) have far more data supporting potential CHD outcomes benefits, compared to virtually no CHD outcome data regarding changes in lipoprotein particle size.

If therapy with a cholesterol-lowering drug can shift patients to Pattern B, what are the potential mechanisms? Impaired LDL clearance is one of the proposed mechanisms accounting for the potential increased atherogenicity of smaller, more dense LDL particles[17]. Studies using LDL particle subspecies from normolipidemic subjects suggest that the small, dense LDL subspecies have lower receptor binding activity compared with the larger, more buoyant LDL particles[17]. It might therefore be expected that, when hepatic LDL receptors are unregulated (as occurs with both ezetimibe and statins) [37], this would preferentially remove the larger circulating LDL particles, which are most easily cleared. So while the cholesterol from all LDL subclasses is reduced, the cholesterol carried by the larger particles may be preferentially reduced, resulting in a disproportionate number of small, denser particles left uncleared, and thus resulting in a post-treatment shift to Pattern B. It is also possible that once LDL-C is rendered below 100 mg/dL, the incorporation of cholesterol into larger LDL particles is reduced[35,38].

This analysis is limited by the utilization of only one methodology to assess lipoprotein particle size and pattern shift. However, this report is one of only a few to report lipoprotein particle size with statin therapy, with or without ezetimibe. While the results of the current analysis are generally consistent with previous reports, this study is at variance with some other reports regarding the effects of statin and ezetimibe upon Pattern B. This may be due to differences in the nature or number of study participants, differences in lipid entry criteria, differences in the lipid-altering agents being assessed, the presence or absence of a control group, and the varied methodologies used to assess particle size and LDL subclasses. However, it is also possible that the variance of this report from some other prior reports is due to publishing bias against prior reporting what might be perceived as "negative" data, or because the effects of statin therapy upon Pattern B is thought to be of questionable clinical significance. This latter explanation is supported by the relative lack of available literature regarding the effects of statins upon Pattern B, relative to the vast amount of published literature on the effects of statins on other lipid parameters. Another potential limitation of this study is that baseline lipid values did not represent a treatment-naïve population. Therefore, the results reflect the effects of a change in atorvastatin 40 mg/d (i.e., addition of ezetimibe or increase in atorvastatin to 80 mg) rather than the change from pre-treatment levels. In addition, due to the *post hoc *nature of some of the analyses, no inferential statistics were conducted for change from baseline in pattern shift or in triglyceride subgroups. Finally, this study provides no direct insight into the CHD outcome merits of particle size and pattern analysis, though other data are available demonstrating the cardiovascular benefits of LDL-C, apo B, and non-HDL-C reductions with statin treatment[39-41].

From a clinical standpoint, although a shift to Pattern B may be misinterpreted by some clinicians, others who advocate for "advanced lipid testing" may not interpret a shift to Pattern B as a detrimental finding with cholesterol-lowering therapy. Instead, these clinicians may perceive the persistence or emergence of Pattern B as an indicator to implement additional lipid-altering therapy that might best reduce the prevalence of Pattern B, such as therapeutic use of niacin, omega-3 fatty acids, or fibrates. Having said this, CHD outcomes data do not yet exist in determining the potential efficacy, or cost effectiveness, of a lipid management approach based on effects on particle size and pattern. It is simply unknown how much residual CHD risk can be alleviated through solely altering lipoprotein pattern and size.

## Conclusion

These results suggest that clinicians should be cautious when interpreting post-treatment lipoprotein particle size pattern results. When assessing the efficacy of cholesterol-lowering therapy, including escalation of cholesterol-lowering therapy as demonstrated in this trial, most CHD outcomes evidence suggests that clinicians should focus on more established efficacy parameters, such as the reduction in the cholesterol carried by LDL particles, atherogenic lipoprotein particle concentration (approximated by apo B), and non-HDL-C levels.

## List of abbreviations

ALT: alanine aminotransferase; apo B: apolipoprotein B; AST: aspartate aminotransferase; CHD: atherosclerotic coronary heart disease; CK: creatinine kinase; FDR: false discovery rate; LDL-C: low-density lipoprotein cholesterol; non-HDL-C: non-high-density lipoprotein cholesterol; NMR: nuclear magnetic resonance; ULN: upper limit of normal; VAP: vertical auto profile

## Competing interests

HB has received research grants, consultant/advisory fees, and speaking honoraria from Merck, Schering-Plough and numerous other pharmaceutical companies. LL has received research grants from Merck, MSP, AstraZeneca, and Roche; has served on speaker's bureaus for Merck, MSP, AstraZeneca, and Roche and as a consultant for Merck, MSP, AstraZeneca, Roche, and Solvay. SB, EJ, MEH, AS, and AMT are employees of Merck and may own stock or stock options in the company.

## Authors' contributions

HB conceived, designed or planned the study, interpreted the results, wrote sections of the initial draft, provided substantive suggestions for revision or critically reviewed subsequent iterations of the manuscript and provided patients. SC and LAL conceived, designed or planned the study, interpreted the results, provided substantive suggestions for revision or critically reviewed subsequent iterations of the manuscript and provided patients. EJ, SB, and AS conceived, designed or planned the study, interpreted the results, wrote sections of the initial draft, provided substantive suggestions for revision or critically reviewed subsequent iterations of the manuscript and contributed statistical expertise. MEH and AMT conceived, designed or planned the study, interpreted the results, wrote sections of the initial draft, provided substantive suggestions for revision or critically reviewed subsequent iterations of the manuscript and provided administrative, technical or logistic support. All authors reviewed and approved the final version of the paper.

## References

[B1] Cannon CP Braunwald E McCabe CH Rader DJ Rouleau JL Belder R Joyal SV Hill KA Pfeffer MA Skene AM Intensive versus moderate lipid lowering with statins after acute coronary syndromes N Engl J Med 2004 350 1495 1504 10.1056/NEJMoa040583 15007110

[B2] LaRosa JC Grundy SM Waters DD Shear C Barter P Fruchart JC Gotto AM Greten H Kastelein JJ Shepherd J Intensive lipid lowering with atorvastatin in patients with stable coronary disease N Engl J Med 2005 352 1425 1435 10.1056/NEJMoa050461 15755765

[B3] Pedersen TR Faergeman O Kastelein JJ Olsson AG Tikkanen MJ Holme I Larsen ML Bendiksen FS Lindahl C Szarek M High-dose atorvastatin vs usual-dose simvastatin for secondary prevention after myocardial infarction: the IDEAL study: a randomized controlled trial JAMA 2005 294 2437 2445 10.1001/jama.294.19.2437 16287954

[B4] Ingelsson E Schaefer EJ Contois JH McNamara JR Sullivan L Keyes MJ Pencina MJ Schoonmaker C Wilson PW D'Agostino RB Clinical utility of different lipid measures for prediction of coronary heart disease in men and women JAMA 2007 298 776 785 10.1001/jama.298.7.776 17699011

[B5] Mudd JO Borlaug BA Johnston PV Kral BG Rouf R Blumenthal RS Kwiterovich PO Jr Beyond low-density lipoprotein cholesterol: defining the role of low-density lipoprotein heterogeneity in coronary artery disease J Am Coll Cardiol 2007 50 1735 1741 10.1016/j.jacc.2007.07.045 17964036

[B6] El Harchaoui K van der Steeg WA Stroes ES Kuivenhoven JA Otvos JD Wareham NJ Hutten BA Kastelein JJ Khaw KT Boekholdt SM Value of low-density lipoprotein particle number and size as predictors of coronary artery disease in apparently healthy men and women: the EPIC-Norfolk Prospective Population Study J Am Coll Cardiol 2007 49 547 553 10.1016/j.jacc.2006.09.043 17276177

[B7] Hammond MG Fisher WR The characterization of a discrete series of low density lipoproteins in the disease, hyper-pre-beta-lipoproteinemia. Implications relating to the structure of plasma lipoproteins J Biol Chem 1971 246 5454 5465 4999356

[B8] Jungner I Sniderman AD Furberg C Aastveit AH Holme I Walldius G Does low-density lipoprotein size add to atherogenic particle number in predicting the risk of fatal myocardial infarction? Am J Cardiol 2006 97 943 946 10.1016/j.amjcard.2005.10.062 16563891

[B9] Kulkarni KR Cholesterol profile measurement by vertical auto profile method Clin Lab Med 2006 26 787 802 10.1016/j.cll.2006.07.004 17110240

[B10] Freedman DS Otvos JD Jeyarajah EJ Barboriak JJ Anderson AJ Walker JA Relation of lipoprotein subclasses as measured by proton nuclear magnetic resonance spectroscopy to coronary artery disease Arterioscler Thromb Vasc Biol 1998 18 1046 1053 9672064 10.1161/01.atv.18.7.1046

[B11] Lamarche B Lemieux I Despres JP The small, dense LDL phenotype and the risk of coronary heart disease: epidemiology, patho-physiology and therapeutic aspects Diabetes Metab 1999 25 199 211 10499189

[B12] Mack WJ Krauss RM Hodis HN Lipoprotein subclasses in the Monitored Atherosclerosis Regression Study (MARS). Treatment effects and relation to coronary angiographic progression Arterioscler Thromb Vasc Biol 1996 16 697 704 8963728 10.1161/01.atv.16.5.697

[B13] Rosenson RS Otvos JD Freedman DS Relations of lipoprotein subclass levels and low-density lipoprotein size to progression of coronary artery disease in the Pravastatin Limitation of Atherosclerosis in the Coronary Arteries (PLAC-I) trial Am J Cardiol 2002 90 89 94 10.1016/S0002-9149(02)02427-X 12106834

[B14] Stampfer MJ Krauss RM Ma J Blanche PJ Holl LG Sacks FM Hennekens CH A prospective study of triglyceride level, low-density lipoprotein particle diameter, and risk of myocardial infarction JAMA 1996 276 882 888 10.1001/jama.276.11.882 8782637

[B15] Rubins HB Robins SJ Collins D Fye CL Anderson JW Elam MB Faas FH Linares E Schaefer EJ Schectman G Gemfibrozil for the secondary prevention of coronary heart disease in men with low levels of high-density lipoprotein cholesterol N Engl J Med 1999 341 410 8 10.1056/NEJM199908053410604 10438259

[B16] El Harchaoui K Arsenault BJ Franssen R Despres JP Hovingh GK Stroes ES Otvos JD Wareham NJ Kastelein JJ Khaw KT High-density lipoprotein particle size and concentration and coronary risk Ann Intern Med 2009 150 84 93 19153411 10.7326/0003-4819-150-2-200901200-00006

[B17] Nigon F Lesnik P Rouis M Chapman MJ Discrete subspecies of human low density lipoproteins are heterogeneous in their interaction with the cellular LDL receptor J Lipid Res 1991 32 1741 1753 1770294

[B18] Anber V Griffin BA McConnell M Packard CJ Shepherd J Influence of plasma lipid and LDL-subfraction profile on the interaction between low density lipoprotein with human arterial wall proteoglycans Atherosclerosis 1996 124 261 271 10.1016/0021-9150(96)05842-X 8830938

[B19] Tribble DL Holl LG Wood PD Krauss RM Variations in oxidative susceptibility among six low density lipoprotein subfractions of differing density and particle size Atherosclerosis 1992 93 189 199 10.1016/0021-9150(92)90255-F 1590824

[B20] Robinson JG Smith B Maheshwari N Schrott H Pleiotropic effects of statins: benefit beyond cholesterol reduction? A meta-regression analysis J Am Coll Cardiol 2005 46 1855 1862 10.1016/j.jacc.2005.05.085 16286171

[B21] Leiter LA Bays H Conard S Bird S Rubino J Hanson ME Tomassini JE Tershakovec AM Efficacy and safety of ezetimibe added on to atorvastatin (40 mg) compared with uptitration of atorvastatin (to 80 mg) in hypercholesterolemic patients at high risk of coronary heart disease Am J Cardiol 2008 102 1495 1501 10.1016/j.amjcard.2008.09.076 19026303

[B22] Davidson MH Bays HE Stein E Maki KC Shalwitz RA Doyle R Effects of fenofibrate on atherogenic dyslipidemia in hypertriglyceridemic subjects Clin Cardiol 2006 29 268 273 10.1002/clc.4960290609 16796078 PMC6653967

[B23] Expert panel on detection evaluation and treatment of high blood cholesterol in adults Executive Summary of The Third Report of The National Cholesterol Education Program (NCEP) Expert Panel on Detection, Evaluation, And Treatment of High Blood Cholesterol In Adults (Adult Treatment Panel III) JAMA 2001 285 2486 2497 10.1001/jama.285.19.2486 11368702

[B24] Kulkarni KR Garber DW Marcovina SM Segrest JP Quantification of cholesterol in all lipoprotein classes by the VAP-II method J Lipid Res 1994 35 159 168 8138718

[B25] Benjamini Y Hochberg Y Controlling the False Discovery Rate: A Practical and Powerful Approach to Multiple Testing J R Statist Soc B (Methodological) 1995 57 289 300

[B26] Ikewaki K Terao Y Ozasa H Nakada Y Tohyama J Inoue Y Yoshimura M Effects of atorvastatin on nuclear magnetic resonance-defined lipoprotein subclasses and inflammatory markers in patients with hypercholesterolemia J Atheroscler Thromb 2009 16 51 56 19261998 10.5551/jat.e563

[B27] The Diabetes Atorvastatin Lipid Intervention (DALI) Study Group The effect of aggressive versus standard lipid lowering by atorvastatin on diabetic dyslipidemia: the DALI study: a double-blind, randomized, placebo-controlled trial in patients with type 2 diabetes and diabetic dyslipidemia Diabetes Care 2001 24 1335 1341 10.2337/diacare.24.8.1335 11473066

[B28] Airan-Javia SL Wolf RL Wolfe ML Tadesse M Mohler E Reilly MP Atheroprotective lipoprotein effects of a niacin-simvastatin combination compared to low- and high-dose simvastatin monotherapy Am Heart J 2009 157 687 688 10.1016/j.ahj.2009.01.001 19332196 PMC3088112

[B29] Maki KC McKenney JM Reeves MS Lubin BC Dicklin MR Effects of adding prescription omega-3 acid ethyl esters to simvastatin (20 mg/day) on lipids and lipoprotein particles in men and women with mixed dyslipidemia Am J Cardiol 2008 102 429 433 10.1016/j.amjcard.2008.03.078 18678300

[B30] Rizzo M Berneis K The clinical significance of the size of low-density-lipoproteins and the modulation of subclasses by fibrates Cur Med Res Opin 2007 23 1103 1111 10.1185/030079907X187892 17519077

[B31] Farnier M Roth E Gil-Extremera B Mendez GF Macdonell G Hamlin C Perevozskaya I Davies MJ Kush D Mitchel YB Efficacy and safety of the coadministration of ezetimibe/simvastatin with fenofibrate in patients with mixed hyperlipidemia Am Heart J 2007 153 335 338 10.1016/j.ahj.2006.10.031 17239698

[B32] Tribble DL Farnier M Macdonell G Perevozskaya I Davies MJ Gumbiner B Musliner TA Effects of fenofibrate and ezetimibe, both as monotherapy and in coadministration, on cholesterol mass within lipoprotein subfractions and low-density lipoprotein peak particle size in patients with mixed hyperlipidemia Metabolism 2008 57 796 801 10.1016/j.metabol.2008.01.026 18502262

[B33] Kalogirou M Tsimihodimos V Gazi I Filippatos T Saougos V Tselepis AD Mikhailidis DP Elisaf M Effect of ezetimibe monotherapy on the concentration of lipoprotein subfractions in patients with primary dyslipidaemia Curr Med Res Opin 2007 23 1169 1176 10.1185/030079907X188062 17519084

[B34] Tomassini JE Mazzone T Goldberg RB Guyton JR Weinstock RS Polis A Jensen E Tershakovec AM Effect of ezetimibe/simvastatin compared with atorvastatin on lipoprotein subclasses in patients with type 2 diabetes and hypercholesterolaemia Diabetes Obes Metab 2009 11 855 864 10.1111/j.1463-1326.2009.01061.x 19508464

[B35] Stojakovic T de Campo A Scharnagl H Sourij H Schmolzer I Wascher TC Marz W Differential effects of fluvastatin alone or in combination with ezetimibe on lipoprotein subfractions in patients at high risk of coronary events Eur J Clin Invest 2010 40 187 194 10.1111/j.1365-2362.2009.02249.x 20067513

[B36] Berneis K Rizzo M Berthold HK Spinas GA Krone W Gouni-Berthold I Ezetimibe alone or in combination with simvastatin increases small dense low-density lipoproteins in healthy men: a randomized trial Eur Heart J 2010 31 1633 1639 10.1093/eurheartj/ehq181 20525999

[B37] Bays HE Neff D Tomassini JE Tershakovec AM Ezetimibe: cholesterol lowering and beyond Expert Rev Cardiovasc Ther 2008 6 447 470 10.1586/14779072.6.4.447 18402536

[B38] Le NA Small, dense low-density lipoprotein: risk or myth? Curr Atheroscler Rep 2003 5 22 28 10.1007/s11883-003-0064-4 12562538

[B39] Cannon CP Steinberg BA Murphy SA Mega JL Braunwald E Meta-analysis of cardiovascular outcomes trials comparing intensive versus moderate statin therapy J Am Coll Cardiol 2006 48 438 445 10.1016/j.jacc.2006.04.070 16875966

[B40] Simes RJ Marschner IC Hunt D Colquhoun D Sullivan D Stewart RA Hague W Keech A Thompson P White H Relationship between lipid levels and clinical outcomes in the Long-term Intervention with Pravastatin in Ischemic Disease (LIPID) Trial: to what extent is the reduction in coronary events with pravastatin explained by on-study lipid levels? Circulation 2002 105 1162 1169 10.1161/hc1002.105136 11889008

[B41] Gotto AM Jr Whitney E Stein EA Shapiro DR Clearfield M Weis S Jou JY Langendorfer A Beere PA Watson DJ Relation between baseline and on-treatment lipid parameters and first acute major coronary events in the Air Force/Texas Coronary Atherosclerosis Prevention Study (AFCAPS/TexCAPS) Circulation 2000 101 477 484 10662743 10.1161/01.cir.101.5.477

